# Relationship between physical activity and behaviour challenges of adolescents in South Africa

**DOI:** 10.4102/sajpsychiatry.v29i0.2124

**Published:** 2023-12-18

**Authors:** Kwabena Asare, Vuyokazi Ntlantsana, Karina Ranjit, Andrew Tomita, Saeeda Paruk

**Affiliations:** 1 Discipline of Public Health Medicine, School of Nursing and Public Health, College of Health Sciences, University of KwaZulu-Natal, Durban, South Africa; 2 Discipline of Psychiatry, School of Clinical Medicine, College of Health Sciences, University of KwaZulu-Natal, Durban, South Africa; 3 Centre for Rural Health, School of Nursing and Public Health, College of Health Sciences, University of KwaZulu-Natal, Durban, South Africa; 4 KwaZulu-Natal Research Innovation and Sequencing Platform (KRISP), College of Health Sciences, University of KwaZulu-Natal, Durban, South Africa

**Keywords:** physical activity, strengths and difficulties questionnaire, behavioural challenges, depression, emotinal challenges

## Abstract

**Background:**

Four out of five adolescents worldwide are physically inactive based on recommended standards.

**Aim:**

We determined whether physical activity is associated with lower behavioural challenges in adolescents to promote buy-in from stakeholders.

**Setting:**

KwaZulu-Natal province, South Africa, from January 2020 to March 2020.

**Methods:**

A cross-sectional study was conducted among 187 adolescent learners (12–18 years) from three government schools in KwaZulu-Natal Province, South Africa, from January to March 2020. We fitted linear regression models between the Strengths and Difficulties Questionnaire scores (total, internalising, externalising, and prosocial) and hours of physical activity exposure, adjusting for demographic covariates, and depression history.

**Results:**

The median age was 14.4 years (interquartile range = 1.36) and 75.9% of the participants were females. Overall average and weekday physical activity were each associated with lower total and externalising but higher pro-social scores. Depression was associated with higher inactivity scores (total, internalising and externalising).

**Conclusions:**

The article shows that physical activity can reduce the behavioural and emotional problems in adolescents.

**Contribution:**

Physical activity is critical for a healthy adolescent hood and needs to be actively included in childhood development.

## Introduction

The adolescent period of life, from 10 years to 19 years, where people transition from childhood to adulthood, is a critical phase of human development, particularly for laying the foundations for good health and life outcomes.^1^ As adolescence is a period of rapid physical, cognitive and psychosocial growth,^1^ the related pubertal tempo, which is the rate of transition to adulthood, has implications for adolescent mood and behaviour, often leading to a myriad of emotional, conduct, hyperactivity, peer problem, and anti-social behavioural disorders.^2,3,4^ These can consequently affect adolescent life choices regarding alcohol and illicit drug use, risky sexual practices, and physical activity, with potential consequences for their health and well-being.^4,5,6,7,8^

The behavioural challenges adolescents face are also known to predominantly co-exist with mental health disorders, such as depression,^9,10^ with numerous evidence in the literature reporting a bidirectional association.^5,10,11,12^ Physical activity can play a major role in reducing the occurrence and consequences of behavioural and mental health disorders in children and adolescents.^13^ Physical activity is known to improve brain health, cognition and academic performance, and reduce symptoms of depression in adolescents.^14^ Accordingly, the World Health Organization (WHO) recommends that children and adolescents aged 5 years–17 years should engage in a minimum of 60 min of moderate-to-vigorous physical activity daily, mostly aerobic in nature.^15^ However, global estimates suggest that over 80% of adolescents are physically inactive, based on these guidelines.^5,15^

Additional evidence on how physical activity affects other health-related outcomes is important for creating buy-in from relevant stakeholders. In this study, we investigated the association between physical activity and behavioural problems in South African adolescents.

## Research methods and design

### Study design, setting, participants, and procedure

This cross-sectional study sampled 187 adolescent learners (12 years–18 years) from three government schools in KwaZulu-Natal province, South Africa, from January to March 2020. All learners from Grades 8–11 from these schools were provided with the study information via the teachers to take home to inform their parents. Those who assented (with parents or legal guardian permission) in writing were enrolled into the study. In addition to obtaining written assent, study participants were provided with a mental health resource help sheet that included contact details for mental health services. The Institutional Review Board at a large public university and the provincial Department of Education in South Africa, approved the research.

### Measures

The study outcomes were emotional symptoms, conduct problems, hyperactivity or inattention, peer relationship problems, and prosocial behaviour. Parent and adolescent versions of the strengths and difficulties short (25-item) measure was used to assesses these five emotional and behavioural subscales:^16^ emotional symptoms (five items); conduct problems (five items); hyperactivity or inattention (five items); peer relationship problems (five items); and prosocial behaviour (five items). As a valid and widely used instrument in developing countries^17,18,19^ including South Africa,^20^ the strengths and difficulties questionnaire (SDQ) has been translated and used in isiZulu, the local language of KwaZulu-Natal province, where the study took place. In this investigation, we applied a three-factor sub-scale model (internalising, externalising, and prosocial) rather than the above-mentioned five subscales, based on fit of confirmatory factor analyses undertaken by a South Africa study.^21^ The internalising SDQ problems involved the SDQ’s emotional symptoms and peer relationship problems subscales.^21,22^ The externalising SDQ problems involved the conduct problems and hyperactivity or inattention subscales.^21,22^ Each item was based on a 3-point Likert scale (0 = not true, 1 = somewhat true, and 2 = certainly true), and the total score for each of the three sub-scales were calculated, with higher scores indicating greater internalising and externalising challenges (with exception of prosocial behaviour).

The Children’s Leisure Activities Survey (CLASS) was used to measure physical leisure time activities, and as a valid and reliable instrument,^23,24^ has been used in South Africa.^24^ The questionnaire^25^ asks participants the average time spent on various activities (i.e. swimming, tennis, soccer, walking for leisure, walking as a means of travel to and from school just to name a few) in minutes per day during weekday and weekend days separately (expressed by average hours per day in this study). The socio-demographic details were also collected in our study and included age, gender, race, and educational level, as well as lifetime history of depression (based on self-report).

### Statistical method

Two analyses were undertaken for this investigation. Firstly, a descriptive summary of the socio-demographic and clinical characteristics of our study participants. Secondly, we fitted four separate linear regression models to examine the role of physical activity against the SDQ ([1] total, [2] internalising, [3] externalising, and [4] prosocial behaviour SDQ scores). This was undertaken thrice to examine the role of total, weekend, and weekday physical activity separately. The regression models controlled for age, gender, race, school grade and history of lifetime depression, with the data being analysed using Stata v17.0 (Stata Corporation. Stata statistical software release 17. College Station, Texas: Stata Corporation LP; 2017).

### Ethical considerations

All learners from Grades 8–11 from three government schools were provided with the study information via the teachers to take home to inform their parents. Those who assented (with parents or legal guardian permission) in writing were enrolled into the study. In addition to obtaining written assent, study participants were provided with a mental health resource help sheet that included contact details for mental health services. The study was approved by the University of KwaZulu-Natal Biomedical Research Ethics Committee (BREC/00000503/2019) and the provincial Department of Education. The study was conducted according to acceptable research standards, including having obtained informed consent of study participants.

## Results

### Socio-demographic and clinical characteristics of adolescent learners

A total of 187 students participated in the study and 75% of them were female (*n* = 142, 75.9%) as indicated in Table 1. With a median age of 14.4 (interquartile range [IQR] = 1.36), 26 study participants (14.5%) reported a lifetime history of depression, based on self-report. Regardless of weekday or weekend, the mean physical activity was approximately 2.4 h per day.

**TABLE 1:** Socio-demographic and clinical characteristics of adolescent learners (*N* = 187).

Variable	Overall			
	*n*	%	Mean	s.d.
**Gender**				
Male	42	22.46	-	-
Female	142	75.94	-	-
Other	3	1.60	-	-
**Grade**				
Grade 8	80	42.78	-	-
Grade 9	50	26.74	-	-
Grade 10	11	5.88	-	-
≥ Grade 11	46	24.60	-	-
**Lifetime depression**				
No	153	85.47	-	-
Yes	26	14.53	-	-
**SDQ learner scores**				
SDQ total	-	-	12.85	5.70
SDQ internalising	-	-	6.98	3.28
SDQ externalising	-	-	5.88	3.39
SDQ prosocial	-	-	7.60	1.86
**Physical activity (hours per day)**				
Average physical activity	-	-	2.43	1.94
Weekend physical activity	-	-	2.47	2.71
Weekday physical activity	-	-	2.41	2.05

Note: For age the median = 14.39 and IQR = 1.36.

SDQ, strengths and difficulties questionnaire; s.d., standard deviation; IQR, interquartile range.

### Association between physical activity and strengths and difficulties questionnaire

Our analyses yielded two important results, firstly, we found that history of (lifetime) depression was significantly associated with higher SDQ (total, internalising and externalising) score. Secondly, we found that overall average physical activity (Table 2) and weekday physical activity (Table 3) were significantly associated with improvement in SDQ (i.e. lower total and externalising, but higher prosocial score). No significant association was detected against SDQ internalising score or in the role of weekend physical activity against any aspects of SDQ (Table 4).

**TABLE 2:** Association between overall physical activity and strengths and difficulties questionnaire scores.

Variable	SDQ total			SDQ internalising			SDQ externalising			SDQ prosocial		
	β Coef.	s.e.	*p*	β Coef.	s.e.	*p*	β Coef.	s.e.	*p*	β Coef.	s.e.	*p*
**Age**	0.34	0.53	0.526	0.09	0.31	0.772	0.25	0.34	0.461	−0.14	0.19	0.474
**Gender**												
[Male]	-	-	-	-	-	-	-	-	-	-	-	-
**Female**	0.89	0.92	0.336	0.89	0.53	0.097	0.00	0.59	0.996	0.30	0.33	0.360
Other	0.10	3.05	0.973	0.20	1.76	0.907	−0.10	1.94	0.959	−0.26	1.09	0.811
**Grade**												
[Grade 8]	-	-	-	-	-	-	-	-	-	-	-	-
Grade 9	−2.52	1.07	0.020	−0.95	0.62	0.124	−1.57	0.68	0.023	−0.21	0.38	0.581
Grade 10	−0.26	1.97	0.897	0.02	1.14	0.987	−0.27	1.25	0.827	0.70	0.71	0.325
≥Grade 11	−1.23	1.80	0.493	0.49	1.03	0.634	−1.73	1.14	0.132	0.86	0.64	0.185
**Lifet ime depression**												
[No]	-	-	-	-	-	-	-	-	-	-	-	-
Yes	7.18	1.12	< 0.001	4.04	0.64	< 0.001	3.14	0.71	< 0.001	−0.03	0.40	0.933
Average physical activity (hours per day)	−0.45	0.20	0.026	−0.21	0.12	0.067	−0.24	0.13	0.065	0.20	0.07	0.007

SDQ, strengths and difficulties questionnaire; s.e., standard error.

**TABLE 3:** Association between weekday physical activity and strengths and difficulties questionnaire.

Variable	SDQ total			SDQ Internalising			SDQ externalising			SDQ Prosocial		
	β Coef.	s.e.	*p*	β Coef.	s.e.	*p*	β Coef.	s.e.	*p*	β Coef.	s.e.	*p*
Age	0.36	0.53	0.500	0.097	0.307	0.753	0.26	0.34	0.436	−0.15	0.19	0.444
**Gender**												
[Male]	-	-	-	-	-	-	-	-	-	-	-	-
Female	0.85	0.93	0.362	0.883	0.535	0.101	−0.03	0.59	0.953	0.32	0.33	0.329
Other	0.02	3.05	0.995	0.187	1.762	0.916	−0.17	1.94	0.931	−0.22	1.09	0.840
**Grade**												
[Grade 8]	-	-	-	-	-	-	-	-	-	-	-	-
Grade 9	−2.46	1.07	0.023	−0.924	0.618	0.137	−1.54	0.68	0.025	−0.24	0.38	0.536
Grade 10	−0.04	1.97	0.985	0.119	1.138	0.917	−0.16	1.25	0.900	0.60	0.71	0.395
≥Grade 11	−1.13	1.79	0.529	0.579	1.035	0.577	−1.71	1.14	0.134	0.82	0.64	0.205
**Lifetime depression**												
[No]	-	-	-	-	-	-	-	-	-	-	-	-
Yes	7.20	1.12	< 0.001	4.058	0.644	< 0.001	3.14	0.71	< 0.001	−0.04	0.40	0.921
Weekday physical activity:	−0.41	0.19	0.032	−0.165	0.109	0.132	−0.24	0.12	0.043	0.18	0.07	0.007

SDQ, strengths and difficulties questionnaire; s.e., standard error.

**TABLE 4:** Association between weekend physical activity and strengths and difficulties questionnaire.

Variable	SDQ total			SDQ internalising			SDQ externalising			SDQ prosocial		
	β Coef.	s.e.	*p*	β Coef.	s.e.	*p*	β Coef.	s.e.	*p*	β Coef.	s.e.	*p*
Age	0.30	0.54	0.573	0.07	0.31	0.827	0.24	0.34	0.491	−0.12	0.19	0.530
**Gender**												
[Male]	-	-	-	-	-	-	-	-	-	-	-	-
Female	1.05	0.93	0.258	0.97	0.53	0.071	0.09	0.59	0.881	0.23	0.33	0.488
Other	0.37	3.07	0.905	0.34	1.76	0.848	0.03	1.95	0.987	−0.38	1.11	0.734
**Grade**												
[Grade 8]	-	-	-	-	-	-	-	-	-	-	-	-
Grade 9	−2.54	1.08	0.020	−0.98	0.62	0.114	−1.55	0.69	0.025	−0.21	0.39	0.594
Grade 10	−0.42	2.00	0.834	−0.13	1.14	0.912	−0.29	1.27	0.818	0.76	0.72	0.291
≥ Grade 11	−0.96	1.81	0.596	0.55	1.03	0.592	−1.52	1.15	0.189	0.73	0.65	0.264
**Lifetime depression**												
[No]	-	-	-	-	-	-	-	-	-	-	-	-
Yes	7.23	1.12	< 0.001	4.06	0.64	< 0.001	3.17	0.72	< 0.001	−0.06	0.40	0.887
Weekend physical activity (hours per day)	−0.21	0.15	0.148	−0.14	0.08	0.089	−0.07	0.09	0.457	0.09	0.05	0.094

SDQ, strengths and difficulties questionnaire; s.e., standard error.

## Discussion

The results of the study revealed that physical activity can reduce emotional and behavioural difficulties during the rapid growth phase of adolescence. Specifically, higher levels of weekday physical activity were associated with fewer internalising and externalising problems of emotional symptoms, conduct problems, hyperactivity or inattention and peer relationship problems, and improved prosocial behaviour in the adolescents. Although we found no significant association between weekend physical activity and SDQ scores, higher overall or weekly physical activity was associated with lower total SDQ and higher prosocial behaviour scores. The authors also found that adolescents with a lifetime history of depression had more emotional and behavioural problems, but there is no association between lifetime history of depression and prosocial behaviour.

Other studies have demonstrated the effects of physical activity in reducing emotional and behavioural problems measured with the SDQ questionnaire in adolescents.^26,27,28^ The authors found some studies that showed an association between concurrent depression^29^ and mental health disorders,^30^ and higher scores on the emotional SDQ sub-scale. Our finding of no association between weekend physical activity and emotional and behavioural problems in adolescents might partly be because most children and adolescents are generally free to engage in physical activities during the weekend as we saw the highest mean hours of physical activity was recorded during the weekend. Therefore, hours of physical activity during the weekend are likely to be similar among children; hence, differences in the overall physical activity are more reflected in extra activities conducted during the weekdays. This is consistent with another recent larger study from the United Kingdom that used objective measures of physical activity but found no association between physical activity and SDQ scores^31^ that may also be because of other confounders, such as the intensity of the physical activity, which needs to be explored.^27^

The study had some limitations. Firstly, the analyses used cross sectional data and thus could not determine the temporal link between physical activity, the emotional and behavioural difficulties, and prosocial behaviour components of the SDQ. Secondly, the sample size might be too small to draw any conclusions, and hence the results might be prone to some level of randomness, as weak statistical associations between overall weekly physical activity and SDQ internalising and externalising scores was found. Thirdly, the authors used subjective measures of depression and self-reported physical activity, which are prone to social desirability biases, potentially underestimating or overestimating the reported outcomes and corresponding associations. As the authors saw, the average hours of weekly physical activity among children and adolescents who participated in our study was twice the recommended minimum 1 h by the WHO. Further research that uses objective measures of physical activity in children is needed to confirm the positive impact of physical activity on emotional and behavioural challenges. Fourthly, the study had a higher number of female than male participants, so we adjusted for sex in the multivariable analysis to minimise any related confounding of the results. However, we cannot rule out the effect of unmeasured confounding; hence, the authors emphasise the importance of studies with balanced distribution of sex and other demographic variables, which are representative of the underlying population.

## Conclusion

In conclusion, the study adds to and strengthens the existing evidence on the potential role of physical activity in preventing emotional and behavioural problems while improving prosocial behaviour in adolescents. The findings provide reassurance that physical activity is an important factor for childhood development and overall well-being. The authors recommend that population level longitudinal household surveys should incorporate physical activity and SDQ measures to help track temporal relationships overtime. Implementation research is also needed to uncover strategies to improve physical activity in adolescents.
